# Double Large Gallstones Ileus: An Unusual Cause of Mechanical Large Bowel Obstruction

**DOI:** 10.7759/cureus.81020

**Published:** 2025-03-22

**Authors:** Ukoha Kalu, Abil Ansari, Gaurav Maheshwari, Sanjay Joshi

**Affiliations:** 1 General and Colorectal Surgery, Medway NHS Foundation Trust, Gillingham, GBR

**Keywords:** cholecystoduodenal fistula, gallstone ileus, mechanical large bowel obstruction, operative management, unusual

## Abstract

Gallstone ileus (GSI) is an unusual complication of cholelithiasis resulting from the formation of a fistula between the gallbladder and the gastrointestinal tract. When a sufficiently large gallstone passes into the bowel, it can obstruct a narrowed segment. The most common site of GSI is the terminal ileum, typically presenting as small bowel obstruction. However, a rare but potentially serious complication of calculous cholecystitis is gallstone sigmoid ileus, which can cause large bowel obstruction. This report presents the case of a 59-year-old woman who exhibited symptoms of large bowel obstruction caused by a large gallstone lodged in the sigmoid colon, along with a simultaneous large gallstone in the cecum, as revealed by multiplanar abdominopelvic CT. Both gallstones had migrated from the gallbladder to the large bowel through a cholecystoduodenal fistula. Initial conservative and endoscopic management attempts were unsuccessful, leading to the need for emergency surgery. Ultimately, the patient made a full recovery.

## Introduction

Gallstone ileus (GSI) of the sigmoid colon is an exceptionally rare cause of large bowel obstruction, most commonly affecting elderly female patients with significant comorbidities. Chronic inflammation between the gallbladder and the bowel, caused by gallstones, can result in necrosis, walled-off abscesses, and fistula formation (cholecystoenteric fistula). Most enteric fistulas form between the gallbladder and the duodenum, with more uncommon occurrences between the gallbladder and the colon [1]. The distal ileum is the most frequent site of gallstone impaction, occurring in approximately 60-85% of cases [1].

In rare cases, a sigmoid GSI develops due to a cholecystocolonic fistula or a cholecystoduodenal fistula, leading to large bowel obstruction when the gallstone becomes trapped in a narrowed segment of the colon. This narrowing can be caused by conditions such as large bowel malignancy or colonic diverticular strictures [2]. Gallbladder fistulae themselves are uncommon, occurring in approximately 1% of biliary tract procedures. Of these, cholecystoduodenal fistulas account for about 70% of cases, cholecystocolonic fistulas for 10-20%, and cholecystogastric fistulas for the remaining cases [3].

Diagnosis of sigmoid colon GSI is often delayed due to the condition’s insidious nature [1,2]. There are currently no established surgical management guidelines for gallstone sigmoid ileus due to its rarity. Treatment approaches are typically individualized and dependent on the expertise of the surgical or endoscopic team involved. Management options range from conservative treatment aimed at allowing the stone to pass naturally to endoscopic retrieval or lithotripsy. When nonoperative measures fail, various surgical procedures, including large bowel resection with anastomosis or stoma formation, may be required. As a result, patient outcomes can vary significantly based on the chosen management strategy [3].

This report describes an unusual case of a female patient presenting with large bowel obstruction caused by GSI of the sigmoid colon, along with a synchronous large gallstone in the cecum. Prompt radiological investigation and timely surgical intervention led to a successful outcome.

## Case presentation

A 59-year-old woman was admitted through the ED with a 48-hour history of colicky lower abdominal pain, absolute constipation, abdominal distension, fever, and bilious vomiting. She had a medical history of sigmoid colon diverticular disease, calculous cholecystitis, type 2 diabetes mellitus, and a high BMI (45.4 kg/m²).

On physical examination, her abdomen was distended with tenderness in the left lower quadrant, but there were no signs of peritonitis. Blood investigations revealed a CRP level of 169.9 mg/L, a white cell count of 14.2 × 10⁹/L, and normal liver function, electrolyte, and amylase levels.

**Figure 1:**
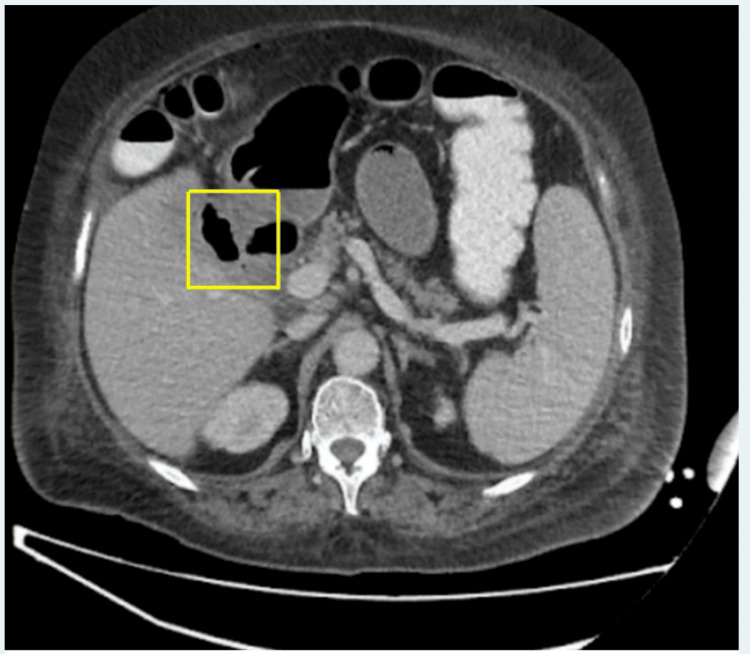
Axial CT image of the abdomen highlighting the cholecystoduodenal fistula, indicated by a yellow rectangular box Air is visible within the thickened gallbladder.

A contrast-enhanced CT scan of the abdomen and pelvis (CTAP) showed extensive pneumobilia, a nondilated common bile duct, and a collapsed gallbladder with significant wall thickening between the second part of the duodenum and the gallbladder, indicating the site of the cholecystoduodenal fistulation, as shown in Figure 1.

**Figure 2:**
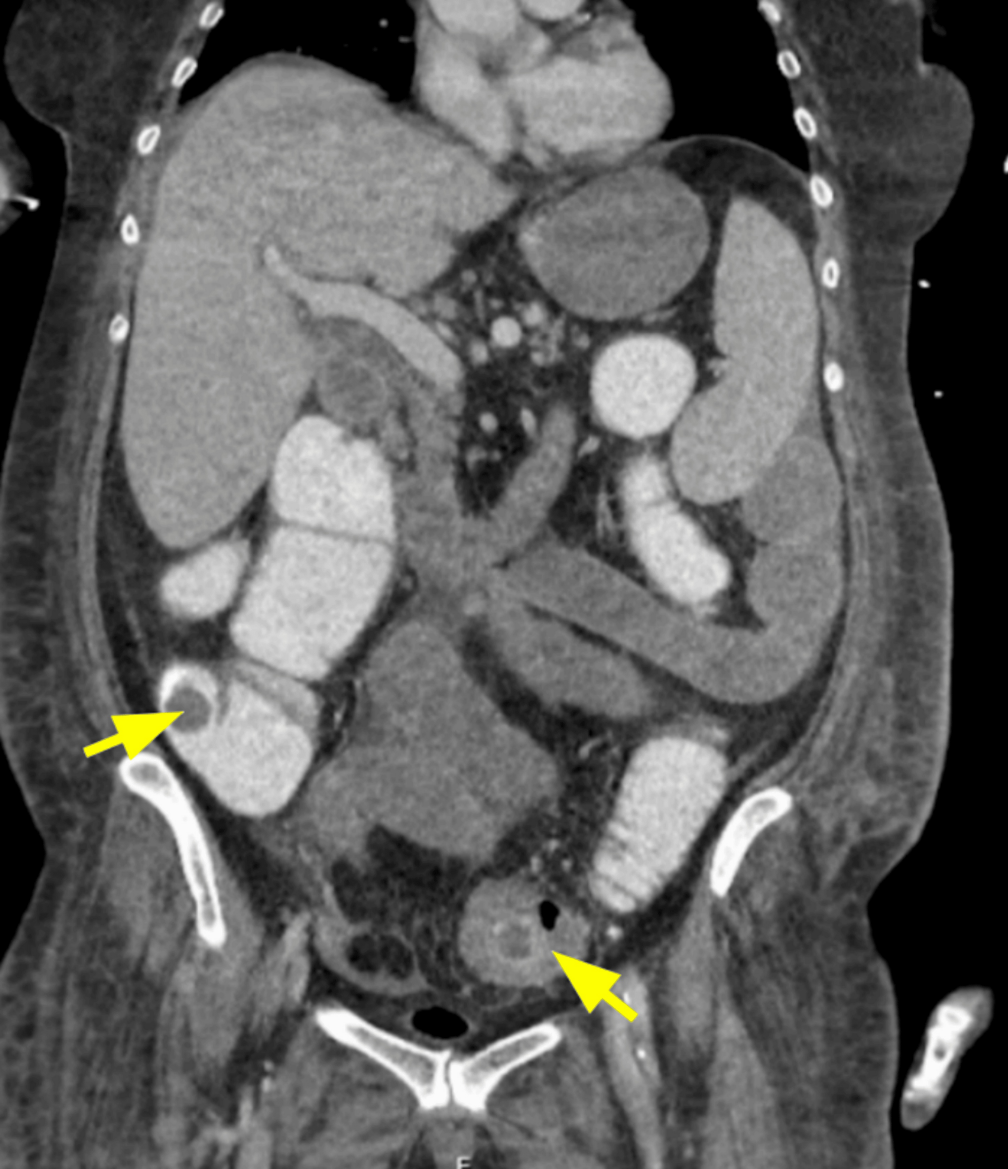
Coronal CT image of the abdomen and pelvis showing synchronous gallstones in the cecum and sigmoid colon, indicated by yellow arrows

The coronal view of the contrast-enhanced CTAP revealed synchronous gallstones in the cecum and the distal sigmoid colon, as demonstrated in Figure 2.

**Figure 3:**
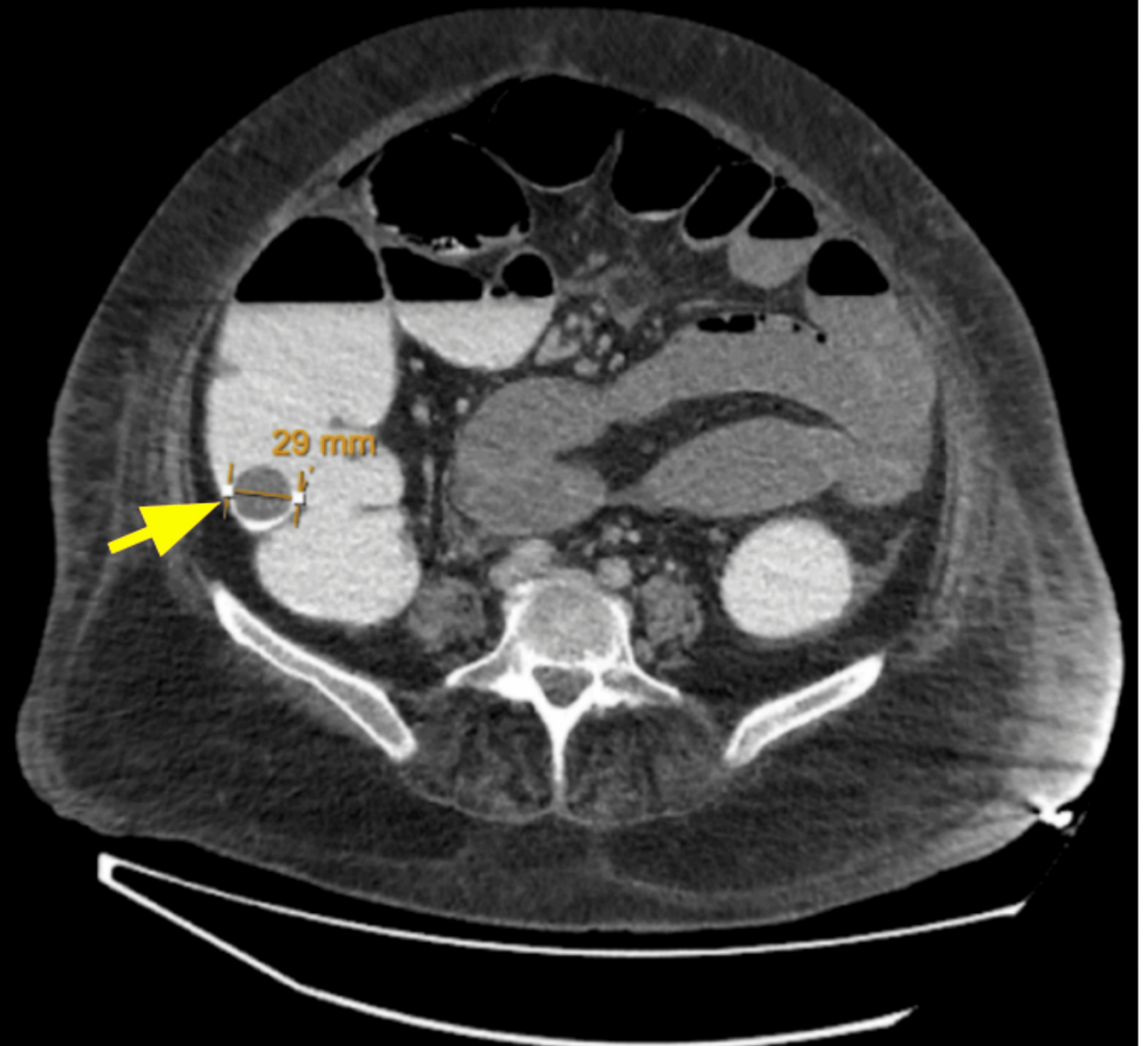
Axial CT image of the abdomen showing a 29 mm gallstone in the cecum, indicated by a yellow arrow

The axial CTAP image, as shown in Figure 3, demonstrated a dilated large bowel loop with a 29 mm gallstone located in the cecum. 

**Figure 4:**
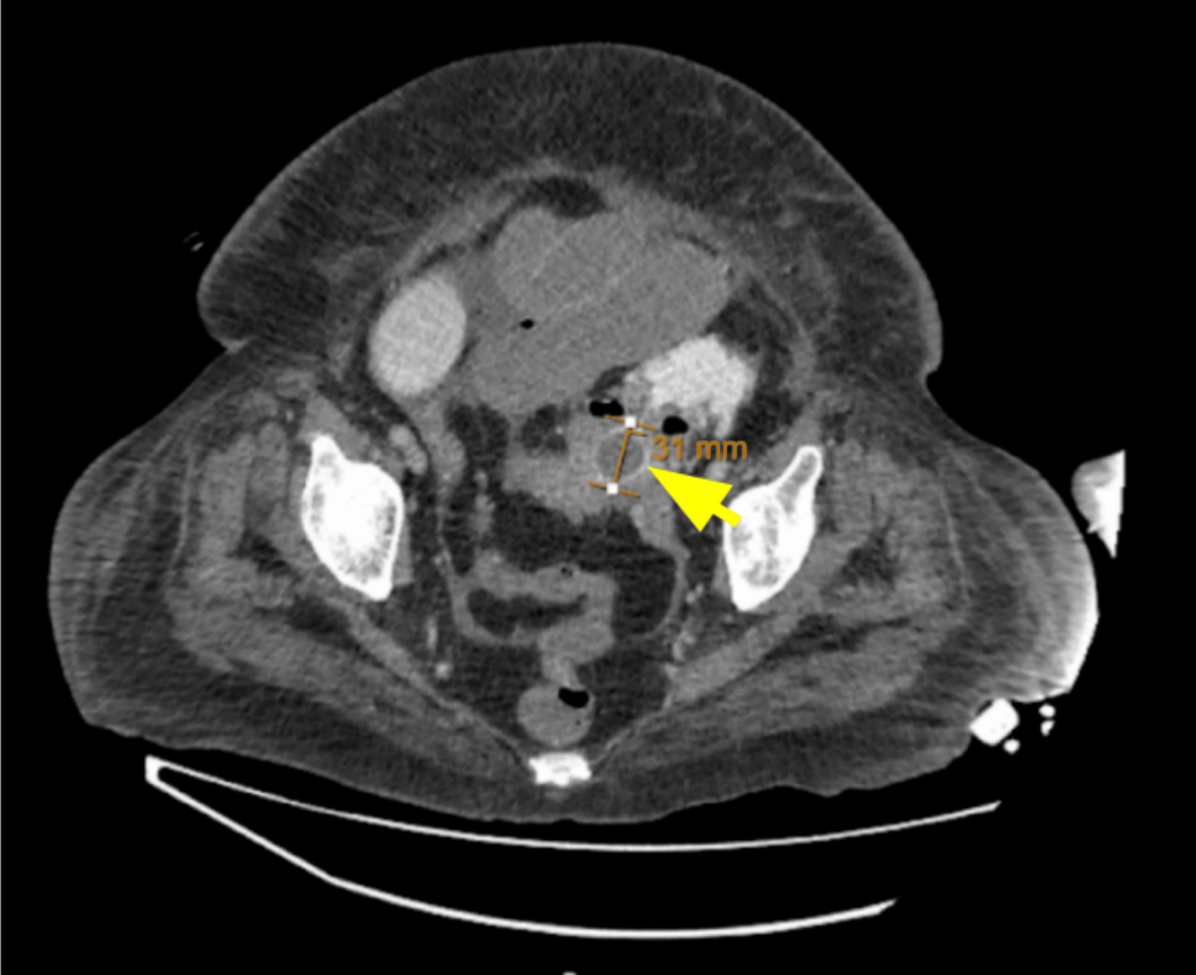
Axial CT image of the abdomen and pelvis showing an impacted 31 mm gallstone in the sigmoid colon, indicated by a yellow arrow

The CTAP image revealed sigmoid colon diverticulitis with mural wall thickening and an impacted 31 mm gallstone in the sigmoid colon, as shown in Figure 4.

Treatment

The patient initially underwent conservative management, including IV fluids, antibiotics, analgesia, oral Gastrografin, and phosphate enema, to attempt flushing the gallstone. However, this approach was unsuccessful.

Flexible sigmoidoscopy was then performed, successfully passing beyond the diverticular stricture within the distal sigmoid colon. Although the gallstone was dislodged, the diverticular stricture of the distal sigmoid colon prevented its retrieval using an endoscopic snare or basket.

After multiple unsuccessful attempts at endoscopic retrieval and due to the absence of a gallstone lithotripsy service at the authors’ hospital, the patient underwent an emergency laparotomy. A laparoscopic Hartmann’s procedure was not feasible due to the markedly distended abdomen.

During the laparotomy, a localized small perforation associated with sigmoid diverticulitis and fixity was identified, leading to the decision to proceed with a Hartmann’s procedure and retrieval of the stone following sigmoid colonic resection. The cholecystoduodenal fistula connecting the gallbladder and the second part of the duodenum was identified, along with the presence of the cecal stone, detected via palpation.

**Figure 5:**
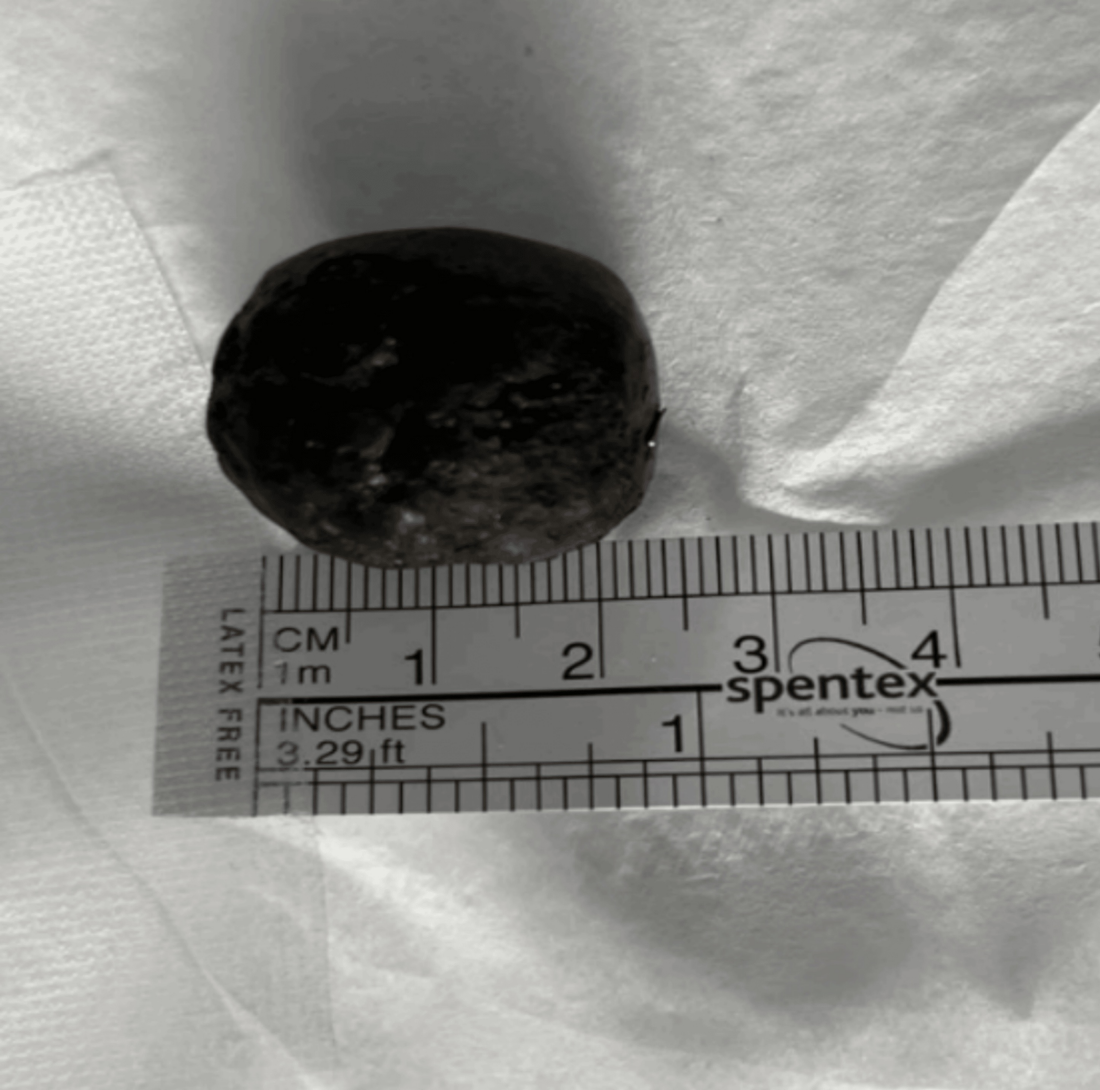
Clinical image of the retrieved gallstone from the cecum, obtained via colostomy on day two following the emergency Hartmann’s procedure

To minimize operative time and reduce the risk of perioperative complications, the decision was made not to repair the cholecystoduodenal fistula and to allow the cecal stone to pass spontaneously. The cecal stone was successfully retrieved on postoperative day two through the end colostomy once the stoma began functioning (Figure 5).

Outcome and follow-up

The patient was discharged home two weeks after surgery and scheduled for a six-week follow-up at the outpatient clinic. She is currently on the waiting list for an elective cholecystectomy and repair of the duodenal fistula, followed by colostomy reversal.

## Discussion

GSI of the sigmoid colon is a rare cause of large bowel obstruction, accounting for only about 2-8% of GSI cases [1]. It typically occurs in elderly patients with multiple comorbidities, with the average age of those affected being around 82 years [3]. However, the patient in this case was notably younger.

Gallstone colonic obstruction usually results from large gallstones greater than 2 cm in diameter that migrate into the colon through a cholecystocolonic fistula. This fistula commonly forms due to recurrent episodes of chronic cholecystitis and adhesions between the gallbladder and the colon, most often at the hepatic colonic flexure [3]. The gallstone may then obstruct the distal sigmoid colon, particularly when there is pathological narrowing caused by conditions such as colonic diverticular disease, malignancy, or previous pelvic irradiation [4].

In this case, two large gallstones migrated through a cholecystoduodenal fistula, as demonstrated by the CTAP images. The stones successfully passed through the ileocecal valve into the cecum, with the most distal stone causing obstruction at the narrowed sigmoid colon, while the proximal stone remained in the cecal lumen, potentially poised to cause further mechanical obstruction at the same narrowed segment.

Patients with sigmoid colon GSI generally present with symptoms consistent with large bowel obstruction, such as colicky abdominal pain and abdominal distension. In some cases, features of ascending cholangitis may also be present. Similar to typical GSI of the small bowel, contrast-enhanced CTAP often reveals a characteristic triad: bowel obstruction, pneumobilia, and ectopic gallstones [3-5]. CTAP imaging is particularly useful for identifying the site of fistulation, the point of obstruction, and the presence of additional gallstones elsewhere in the bowel lumen [5,6], as demonstrated in this case.

Approximately 93% of patients with GSI require endoscopic and/or surgical intervention, while only about 7% are successfully managed through nonoperative methods such as endoscopic retrieval or lithotripsy [7]. Although endoscopic extraction of colonic gallstones has been successful in a few cases, it is generally ineffective when dealing with relatively large stones and a narrowed colonic lumen caused by diverticular strictures, as seen in this patient [6-8].

Emergency surgical management of colonic GSI is generally similar to that of small bowel GSI, involving proximal enterolithotomy to remove the impacted stone and subsequent repair of the enterotomy [9-12]. Definitive treatment involves elective cholecystectomy and repair of the duodenal or colonic fistula to prevent further episodes of ascending cholangitis due to the enterobiliary fistula [13]. However, definitive surgery can be challenging in colonic GSI patients, who are often elderly, frail, and burdened with multiple comorbidities.

## Conclusions

This case highlights gallstone sigmoid colon ileus as a rare cause of large bowel obstruction, particularly in patients with chronic calculous cholecystitis. Contrast-enhanced CT of the abdomen and pelvis plays a crucial role in diagnosing this uncommon cause of mechanical large bowel obstruction by accurately identifying the site of obstruction, underlying colonic pathology, enterobiliary fistula, and the presence of synchronous gallstones within the bowel, as demonstrated in this case. Early surgical intervention should be considered if nonoperative management proves unsuccessful.

## References

[1] Carlsson T, Gandhi S (2015). Gallstone ileus of the sigmoid colon: an extremely rare cause of large bowel obstruction detected by multiplanar CT. BMJ Case Rep.

[2] Farkas N, Kaur V, Shanmuganandan A, Black J, Redon C, Frampton AE, West N (2018). A systematic review of gallstone sigmoid ileus management. Ann Med Surg (Lond).

[3] Lobo DN, Jobling JC, Balfour TW (2000). Gallstone ileus: diagnostic pitfalls and therapeutic successes. J Clin Gastroenterol.

[4] Vaughan-Shaw PG, Talwar A (2013). Gallstone ileus and fatal gallstone coleus: the importance of the second stone. BMJ Case Rep.

[5] Lassandro F, Gagliardi N, Scuderi M, Pinto A, Gatta G, Mazzeo R (2004). Gallstone ileus analysis of radiological findings in 27 patients. Eur J Radiol.

[6] Key A, Dawkins A, DiSantis D (2015). Rigler's triad. Abdom Imaging.

[7] Osman N, Subar D, Loh MY, Goscimski A (2010). Gallstone ileus of the sigmoid colon: an unusual cause of large-bowel obstruction. HPB Surg.

[8] Waterland P, Khan FS, Durkin D (2014). Large bowel obstruction due to gallstones: an endoscopic problem?. BMJ Case Rep.

[9] Papavramidis TS, Potsi S, Paramythiotis D (2009). Gallstone obstructive ileus 3 years post-cholecystectomy to a patient with an old ileoileal anastomosis. J Korean Med Sci.

[10] Da Cunha T, Sharma B, Goldenberg S (2021). Colonic gallstone ileus: treatment challenges. Cureus.

[11] Costi R, Randone B, Violi V (2009). Cholecystocolonic fistula: facts and myths. A review of the 231 published cases. J Hepatobiliary Pancreat Surg.

[12] Zulian V, Vasquez G, Feo CV (2013). Unusual presentation and treatment of biliary ileus with long term follow up: case report and review of the literature. Ann Ital Chir.

[13] Van Kerschaver O, Van Maele V, Vereecken L, Kint M (2009). Gallstone impacted in the rectosigmoid junction causing a biliary ileus and a sigmoid perforation. Int Surg.

